# Predictors of multidrug-resistant tuberculosis in a teaching hospital in Ghana: A case-control study

**DOI:** 10.1371/journal.pone.0294928

**Published:** 2023-11-29

**Authors:** Philomina Afful, Godwin Adjei Vechey, Peter Kipo Leta, Foster Bediako Gbafu, Fortress Yayra Aku

**Affiliations:** 1 Department of Epidemiology and Biostatistics, Fred N. Binka School of Public Health, University of Health and Allied Sciences, Hohoe Campus, Hohoe, Ghana; 2 Public Health Unit, Cape Coast Teaching Hospital, Cape Coast, Ghana; 3 Nkoranza South Municipal Health Directorate, Bono East Region, Nkoranza, Ghana; 4 Malaria Research Centre, Agogo, Ashanti Region, Ghana; Management Sciences for Health (MSH), ETHIOPIA

## Abstract

Multidrug-resistant Tuberculosis (MDR-TB) remains a global health concern. The disease results in a prolonged treatment and hence, poses a financial burden to affected individuals and their families. The Ghana National TB Control Programme (NTP) has made extensive efforts to control the menace, however, it remains a concern. This study, therefore, aimed to determine the predictors of multidrug-resistant TB in the Cape Coast Teaching Hospital of Ghana. An unmatched case-control study involving 37 cases and 111 controls was conducted using data of TB cases registered for treatment between January 2018 and December 2020 at the Cape Coast Teaching Hospital. Socio-demographic, individual level and social characteristics information were collected from respondents through telephone surveys, face-to-face interviews and review of records using a structured questionnaire built in the Kobo Collect Toolbox. The data was exported to Stata version 16.0 for analysis. Chi-square test and multiple logistic regression were used to determine the predictors of MDR-TB. Associations were considered statistically significant at a 95% confidence interval with a p-value of less than 0.05. The results revealed that the majority (25 [67.6%]) of MDR-TB cases and controls (76 [68.5%]) were aged 30 years and above with a median age of 36.5 (IQR: 28–50) years for all respondents, while 20 (54.1%) of MDR-TB cases and 33 (29.7%) of controls lived in households with one room residences for their families. The following predictors for MDR-TB were identified: BCG vaccination status (AOR = 0.17,95% CI:0.07–0.45), long distance to health facility (AOR = 4.11, 95% CI: 1.55–10.87), number of rooms in residence (AOR = 0.37,95% CI: 0.14–0.99) and first place of visit upon noticing TB symptom (AOR = 4.22,95% CI:1.31–13.64). Predictors of MDR-TB in the current study were multi-faceted. Measures to control MDR-TB should target socio-demographic, health-seeking behaviour and social-related concerns.

## Introduction

The upsurge in multidrug-resistant Tuberculosis (MDR-TB) has become a major public health challenge globally [1, 2], notably in resource-limited countries and is commonly associated with unsuccessful treatment outcomes [3]. It occurs when the Mycobacterium strain becomes resistant to both Isoniazid (INH) and Rifampicin (RIF), the two powerful anti-TB drugs [4, 5]. The management and treatment of MDR-TB are complex, and it is hard to achieve favourable treatment outcomes as compared to drug-sensitive TB, even under optimal circumstances [6–8]. The treatment is usually prolonged with a minimum duration of 24 months [9]. In addition inadequate treatment of MDR-TB could lead to worse patient outcomes, including increasing the danger of extensive drug resistance [10].

According to the 2020 World Health Organisation (WHO) Global TB report, 206, 030 MDR-TB cases were recorded in 2019, indicating an increase of 10% from what was previously reported in 2018 (186,882) [11]. In Africa, the report revealed that 2.6% of new and 11% of previously treated cases were estimated to have MDR-TB/RIF-resistant TB (RR-TB) [12]. A cross-sectional study conducted among MDR-TB-suspected patients in Addis Ababa, Ethiopia, reported the prevalence of MDR-TB to be 39.4% with more than 58% of these patients being resistant to all first-line TB drugs [2].

In Ghana, a national drug resistance survey to investigate the level and patterns of resistance to first-line TB drugs reported that, resistance to INH and RIF, the most effective anti-TB drugs, was 3.2% [12]. Furthermore, Davies-Teye et al. [13] reported a cumulative incidence of MDR-TB in the Greater Accra Region of 1.4/100 000 population, with a case fatality rate of 14%.

In the Cape Coast Teaching Hospital of Ghana, a review of annual reports indicated that, in 2015, 0.4% of TB patients tested positive for MDR-TB, with 1.1%, 1.3%, 2.7% and 2.5% testing positive for 2016, 2017, 2018 and 2019, respectively. This data projects an increasing trend of MDR-TB cases over the period under review [14]. The situation jeopardises the gains made by the joint efforts of the Ghana National TB Control Programme (NTP) and other stakeholders in the control of TB [15]. The consequences are adverse, including notably high treatment costs, relapses and mortalities [16]. Identifying context-specific predictors of MDR-TB would be useful to the NTP and relevant stakeholders to avert its occurrence and improve the treatment success rate of patients with MDR-TB. This study therefore, investigated the predictors of MDR-TB in the Cape Coast Teaching Hospital of Ghana.

## Methods

### Study design and sample

An unmatched case-control study was conducted to assess the predictors of MDR-TB in the Cape Coast Teaching Hospital. Registered TB patients diagnosed as having MDR-TB served as cases while TB patients without MDR-TB served as controls. The case-control study design was appropriate, as it allowed for predictors of MDR-TB to be determined. The sample size was calculated using the online OpenEpi sample size calculator, based on the following assumptions:

- 80% power at 95% confidence interva1
- 1:3 ratio (case to control)
- percent of control exposed 35.3%, percent of cases exposed 61.5% based on a previous study in Ethiopia [17].

This resulted in a minimum sample size of 147 (37 cases and 110 controls). However, a total sample size of 148 was used for this study.

### Study setting

The study was conducted in the Cape Coast Teaching Hospital, located in the central region of Ghana. The hospital is currently a four hundred (400) bed capacity referral hospital situated at the northern part of Cape Coast. It was among the first of series of ultra-modern regional hospitals established by the ministry of health and later transformed into a teaching hospital with the inception of the medical sciences programme at the university of Cape Coast. The hospital is now a tertiary level hospital offering several services including: medical, surgical, laboratory, maternal and child health, reproductive health and public health services.

### Inclusion and exclusion criteria

This study enrolled TB patients registered at the Cape Coast Teaching Hospital between January 2018 and December 2020 who had either completed or were still receiving treatment. Cases were included if they were registered TB cases resistant to RIF per the GeneXpert results and consented to be part of the study. Controls were included if they were registered TB cases, were drug-sensitive based on the results from the GenXpert platform and consented to participate in the study. Participants who met the inclusion criteria but had emergency and complex medical conditions requiring critical or urgent attention were excluded from the study.

### Sampling

The TB register for the period January 2018 to December, 2020 was reviewed to identify MDR-TB and non MDR-TB cases. Thirty-seven (37) MDR-TB cases, were identified over the period under review and were purposively selected. To obtain the 1:3 ratio of cases: controls, 111 corresponding controls were selected using a simple random sampling method.

### Case definition

MDR-TB cases were defined as bacteriologically confirmed TB cases that were detected as RIF-resistant by the GeneXpert technique. Controls referred to bacteriologically confirmed TB cases that were sensitive to first-line TB drugs.

### Data collection

Data were collected through telephone surveys (that lasted an average of 20 minutes), and records were reviewed for all selected cases and controls who had completed treatment during the study period. For participants who were still receiving treatment during the study period, a face-to-face interview and records review were conducted during their appointment at the clinic. Three Directly Observed Therapy (DOT) providers who regularly attended to persons with TB at the clinic were trained to conduct the interviews. Data were collected on demographic, individual and social characteristics that contribute to MDR-TB for both MDR-TB cases and controls using a structured questionnaire between July and September, 2021. The questionnaire was pre-tested, which ensured the identification and correction of possible errors and ambiguities in the questionnaire as part of quality control measures. All data were anonymized before analysis.

### Data analysis

Data were entered into Microsoft Excel and checked for completeness to avoid missing data. Interviewer codes of data collectors on the questionnaires and telephone numbers of respondents allowed for clarification and follow-up of any missing data. Data was then exported into STATA software version 16.0 for analysis. Descriptive analysis was performed by computing frequencies and proportions. For inferential analysis, chi-square test was performed to test for an association between MDR-TB cases and respondent characteristics. Multiple logistic regression was performed to compute the odds ratio, to test for predictors of MDR-TB cases, and the statistical significance level was determined at 95% confidence interval and p value of 0.05.

### Ethical approval

Ethical approval for the study was obtained from the University of Health and Allied Sciences Ethical Review Committee (UHAS-REC) with identification number UHAS REC A.10[70] 20–21 while written permission was sought from the management of the Cape Coast Teaching Hospital. Informed consent was obtained from all respondents prior to data collection and patients’ right to voluntarily participate, withdraw and skip questions were respected. Data were coded to ensure anonymity and responses were only accessible to the research team to ensure confidentiality. Respondents were assured that responses regarding default or non-adherence or otherwise would not affect the care they will receive from the healthcare system in the future. All face-to-face interviews were conducted under strict observation of the COVID-19 protocol.

## Results

### Socio-demographic characteristics of respondents

Of the total of 37 cases and 111 controls included in the study, the median age was 36.5 (IQR: 28–50). More than half of the MDR-TB cases (22 representing 59.5%), and controls (66 representing 59.5%) were males and females respectively. Majority (25 [67.6%]) of the MDR-TB cases and 76 (68.5%) of the controls were 30 years and above. Most of the MDR-TB cases (32 [86.5%]) had attained formal education whiles 97 (87.4%) controls also had attained formal education. Twenty-three (62.2%) of the MDR-TB cases and 62 (55.9%) of the controls were married. In addition, 7 (18.9%) cases and 18 (16.2%) controls were civil servants. Whiles 20 (54.1%) cases and 33 (29.7%) controls lived in a single-room residence with their families, most of the cases (23 [62.2%]) had a family size of three or less, with 54 (48.7%) of the controls having a family size of three or less. Twenty-nine (78.4%) cases and 63 (56.8%) controls had an average monthly income below GHC500 (Table 1).

**Table 1:** Socio-demographic characteristics of MDR-TB cases and their controls.

Variables	Cases(N = 37)	Controls(N = 111)
	n (%)	n(%)
**Sex**		
Female	15(40.5)	45(40.5)
Male	22(59.5)	66(59.5)
**Age group(median 37(IQR:28–50)years**		
<30years	12(32.4)	35(31.5)
≥30years	25(67.6)	76(68.5)
**Educational status**		
Formal education	32(86.5)	97(87.4)
Non-formal education	5(13.5)	14(12.6)
**Marital status**		
Single	14(37.8)	49(44.1)
Married	23(62.2)	62(55.9)
**Occupation**		
Civil servant	7(18.9)	18(16.2)
Non civil servant	30(81.1)	93(83.8)
**Number of rooms in the residence**		
1	20(54.1)	33(29.7)
>1	17(45.9)	78(70.3)
**Family size**		
≤3	23(62.2)	54(48.6)
≥4	14(37.8)	57(51.4)
**Average monthly income**		
Above GHC500	8(21.6)	48(43.2)
Below GHC500	29(78.4)	63(56.8)

### Individual factors of MDR-TB cases and their controls

Twenty-two (59.5%) cases and 47 (42.3%) controls reported they would visit the health facility upon suspicion of a TB sign with the reason of getting effective treatment. Twenty-five (67.6%) cases and 56 (50.5%) controls revealed that, the attitude of health workers during care was friendly. A majority (26 [70.3%]) of the cases stated that, they traveled more than 5km from their homes to reach the facility, while only 37 (33.3%) controls stated to have traveled the same distance to the facility. Also, 31 (83.8%) of cases and 103 (92.8%) controls indicated they had taken all prescribed drugs. While more than half (21 [56.8%]) of the cases indicated they did not receive the BCG vaccine, only 19 (17.1%) of the controls received the BCG vaccine (Table 2).

**Table 2:** Individual factors of cases and controls.

Variable	Cases(N = 37)	Controls(N = 111)
	n(%)	n(%)
**First place of visit upon the first sign of TB**		
Drug store	7(18.9)	50(45.1)
Health facility	30(81.1)	61(54.9)
**Reason for the choice of the place of visit**		
Easily accessible	15(40.5)	64(57.7)
Effective treatment	22(59.5)	47(42.3)
**The attitude of health workers during care**		
Encouraging	12(32.4)	55(49.5)
Friendly	25(67.6)	56(50.5)
**Distance of facility from home**		
≤5km	11(29.7)	74(66.7)
>5km	26(70.3)	37(33.3)
**Taken all prescribed drugs**		
No	6(16.2)	8(7.2)
Yes	31(83.8)	103(92.8)
**Took BCG vaccine**		
Not vaccinated	21(56.8)	19(17.1)
Yes, vaccinated	16(43.2)	92(82.9)
**Ever smoked cigarette**		
Ever smoked	6(16.2)	27(24.3)
Never smoked	31(83.8)	84(75.7)

### Social characteristics of respondents

Twenty-six (70.3%) of the cases reported that they faced challenges receiving support from their neighbours because of TB, and about half (56 [50.5%]) of the controls also affirmed this. Most (24 [66.7%]) cases stated that they were not afraid of losing friends or their jobs due to their conditions; however, more than half (55.5%) of the controls were afraid of losing friends or their jobs because of TB. Likewise, ten (27.8%) cases and 33 (29.7%) controls reported experiencing some form of stigmatization due to their condition, and over half (20 [54.0%]) of the cases stated that, they hid their condition from society (Table 3).

**Table 3:** Social characteristics of cases and controls.

Variable	Cases (N = 37)	Controls(N = 111)
	n(%)	n(%)
**Ease of obtaining assistance from neighbours**		
Yes	11(29.7)	55(49.6)
No	26(70.3)	56(50.4)
**Experienced stigmatisation from TB**		
Yes	10(27.8)	33(29.7)
No	26(72.2)	78(70.3)
**Fear of loss of friends and job**		
Yes	12(33.3)	61(55.5)
No	24(66.7)	49(44.5)
**TB status is confidential**		
Yes	20(54.0)	80(72.1)
No	17(46.0)	31(27.9)

### Predictors of MDR-TB

Table 4 describes the predictors of MDR-TB. The unadjusted logistic regression analysis showed that the number of rooms in residence [cOR: 0.36 (CI, 0.17–0.77), p = 0.009], average monthly income [cOR: 2.76 (CI, 1.16–6.58), p = 0.022], the first place of visit upon the first sign of TB [cOR: 3.51 (CI,1.42–8.67), p = 0.006], distance of the facility from home [cOR: 4.72 (CI, 2.11–10.60), p<0.001], BCG vaccination [0.16 (0.07–0.36), <0.001], ease of obtaining help from neighbours [2.32 (1.05–5.15), 0.038], fear of losing friends/jobs [2.49 (1.13–5.48), 0.023] and hiding their TB status from others [2.19 (1.02–4.73), 0.045] were significantly (p < 0.05) associated with MDR-TB status.

**Table 4:** Predictors of MDR-TB cases.

Variable	Cases (n = 37)	Controls (n = 111)	Chi, p-value	COR (95% CI), p-value	AOR (95% CI), p-value
**Sex**			0.00, 1.000		
Female	15 (40.5)	45 (40.5)			
Male	22 (59.5)	66 (59.5)			
**Age group**			0.010, 0.919		
<30years	12 (32.4)	35 (31.5)			
≥30years	25 (67.6)	76 (68.5)			
**Educational status**			0.02, 0.887		
Formal education	32 (86.5)	97 (87.4)			
Non-formal education	5 (13.5)	14 (12.6)			
**Occupation**			0.14, 0.704		
Civil servant	7(18.9)	18 (16.2)			
Non civil servant	30 (81.1)	93 (83.8)			
**Number of rooms in residence**			7.14, **0.008**		
1	20 (54.1)	33 (29.7)		ref.	ref.
>1	17 (45.9)	78 (70.3)		0.36 (0.17–0.77), **0.009**	0.37 (0.14–0.99), **0.047**
**Family size**			2.03, 0.154		
≤3	23 (62.2)	54 (48.6)			
≥4	14 (37.8)	57 (51.4)			
**Average monthly income**			5.52, **0.019**		
Above GHC500	8 (21.6)	48 (43.2)		ref.	
Below GHC500	29 (78.4)	63 (56.8)		2.76 (1.16–6.58), **0.022**	
**First place of visit upon first sign of TB**			7.99, **0.005**		
Drug store	7 (18.9)	50 (45.1)		ref.	ref.
Health facility	30 (81.1)	61 (54.9)		3.51 (1.42–8.67), **0.006**	4.22 (1.31–13.64), **0.016**
**Reason for visiting the place**			3.27, 0.071		
Easily accessible	15 (40.5)	64 (57.7)			
Effective treatment	22 (59.5)	47 (42.3)			
**Attitude of healthcare provider**			3.28, 0.070		
Unfriendly	12 (32.4)	55 (49.5)			
Friendly	25 (67.6)	56 (50.5)			
**Distance of facility from home**			15.49, <**0.001**		
≤5km	11 (29.7)	74 (66.7)		Ref.	Ref.
>5km	26 (70.3)	37 (33.3)		4.72 (2.11–10.60), <**0.001**	**4.11 (1.55–10.87), 0.004**
**Taken all prescribed drugs**			2.63, 0.105		
No	6 (16.2)	8 (7.2)			
Yes	31 (83.8)	103 (92.8)			
**BCG vaccination**			22.11, <**0.001**		
Not vaccinated	21 (56.8)	19 (17.1)		ref.	Ref.
Vaccinated	16 (43.2)	92 (82.9)		0.16 (0.07–0.36), <**0.001**	0.17 (0.07–0.45), <**0.001**
**Ease of obtaining assistance from neighbours**			4.41, **0.036**		
Yes	11 (29.7)	55 (49.6)		ref.	
No	26 (70.3)	56 (50.4)		2.32 (1.05–5.15), **0.038**	
**Experience stigmatization of TB**			0.05, 0.823		
Yes	10 (27.8)	33 (29.7)			
No	26 (72.2)	78 (70.3)			
**Fear of losing friends and Job**			5.31, **0.021**		
Yes	12 (33.3)	61 (55.4)		ref.	
No	24 (66.7)	49 (44.6)		2.49 (1.13–5.48), **0.023**	
**TB status to be confidential**			4.11, **0.043**		
Yes	20 (54.1)	80 (72.1)		ref.	
No	17 (45.9)	31 (27.9)		2.19 (1.02–4.73), **0.045**	

The adjusted multivariable logistic regression model revealed that individuals who had more than one room in a household were less likely (AOR = 0.37, 95% CI: 0.14–0.99) to have MDR-TB compared with those with one room per household. The odds of having MDR-TB were lower among those who took the BCG vaccine (AOR = 0.17, 95% CI: 0.07–0.45) compared with those who did not. On individual factors, those who visited a health facility upon the first signs of TB were four times (AOR = 4.22, 95% CI: 1.31–13.64) more likely to have MDR-TB. The long distance between home and health facility was four times (AOR = 4.11, 95% CI: 1.55–10.87) more likely to result in the development of MDR-TB compared with those whose homes were close to the health facility (Table 4).

## Discussion

Globally, the emergence of MDR-TB remains a major public health challenge. Global prevalence among newly and previously treated TB patients is found to be 3.6% and 18% respectively [18, 19]. Therefore, this study sought to determine the predictors of MDR-TB among TB patients registered between January 2018 and December 2020 in the Cape Coast Teaching Hospital. The protective nature of Bacille Calmette-Guerin (BCG) vaccination against developing MDR-TB is consistent with the results of a previous study [20] which reported that 30% of MDR-TB cases and 13% of the control group were unvaccinated with the BCG vaccine suggesting its role in MDR-TB prevention. Another study showed that unvaccinated individuals had an increased risk for MDR-TB [21]. This affirms the benefits of the BCG vaccine incorporated into routine immunisation schedules of several countries.

The current study’s finding that TB patients with multiple rooms within a household had lower odds of developing MDR-TB corroborates with the results of a study conducted in Peru, which reported that substandard housing conditions are indicators of predicted treatment default [22], that could culminate into MDR-TB. It also affirms how poor sanitation increases the risk of TB drug resistance. Good sanitation aids in the reduction of MDR-TB, which is aggravated due to poor hygiene and ventilation [23]. In Ethiopia, it was reported that people who lived in households with only one room were five times more at risk of acquiring MDR-TB than those living in more than one room [24], which is coherent with the finding that there is a high risk related to acquiring resistant strains from infected hosts in crowded places. There is, therefore, the need for education on innovative and context-targeted strategies for persons with TB who may be sharing single rooms with their families and friends [25].

Access to TB services may be limited by distance, especially for the daily DOT with regards to MDR-TB conducted in health facilities. Our findings corroborate with those reported by Iweama et al. [26] in Nigeria, Suliman and colleagues [27] in Malaysia, Peresu and colleagues [28] in South Africa and Pradipta [29] in the Netherlands, which reported long distance as the major risk factor for interruption of treatment among TB patients. Patients who are already burdened by the disease (TB) also have to bear additional costs of transporting themselves to access TB services. This makes them more likely to interrupt treatment leading to MDR-TB [30]. According to Kanwal and Akhtar [31] distance to health facilities was a significant predictor of MDR-TB as observed in the current study. However, a study conducted in Indonesia revealed no statistical association between the distance to health facilities and MDR-TB [32] and this difference might be on account of geographical differences in both studies.

Our study highlights the place of seeking care as an important factor to consider when tackling MDR-TB. This is crucial since it is reported that, several patients visit traditional healers and ingest alternative treatment during MDR-TB therapy, which in some cases lead to their discontinuation from conventional treatment [33]. Over-reliance on traditional healing practices by patients was recognized as a constraint to the standard TB care by both patients and providers. This could account for why MDR-TB is still a burden in most countries, including Ghana though some studies did not report this association [34, 35].

### Limitations

The study had some limitations despite the important findings. Recall bias and the study’s inability to verify some participants’ responses could have resulted in social desirability bias. This was mitigated by using trained directly observed therapy (DOT) providers (with whom registered TB cases are familiar and comfortable with) who conducted the interviews in the same manner for both cases and controls. Furthermore, the design did not allow for establishing a causal-effect relationship between MDR-TB and its predictors. However, the study design and the robust statistical method used generated useful findings that could be generalized in the context of those receiving TB care in the Ghanaian community.

## Conclusion

The study revealed that predictors of MDR-TB are diverse, spanning from socio-demographic characteristics to individual characteristics of persons receiving care for TB. It, therefore, reiterates that MDR-TB requires combined efforts of both patients and care providers, and control measures must be context-oriented. Managers of the TB control programme and other stakeholders are to explore innovative ways of addressing these issues.

## Supporting information

S1 Data (XLSX)
